# Non-South East Asians have a better running economy and different anthropometrics and biomechanics than South East Asians

**DOI:** 10.1038/s41598-022-10030-4

**Published:** 2022-04-15

**Authors:** Aurélien Patoz, Thibault Lussiana, Bastiaan Breine, Cyrille Gindre, Laurent Mourot, Kim Hébert-Losier

**Affiliations:** 1 Institute of Sport Sciences, University of Lausanne, 1015 Lausanne, Switzerland; 2 Research and Development Department, Volodalen Swiss Sport Lab, 1860 Aigle, Switzerland; 3 Research and Development Department, Volodalen, 39270 Chavéria, France; 4 Research Unit EA3920 Prognostic Markers and Regulatory Factors of Cardiovascular Diseases and Exercise Performance, Health, Innovation Platform, University of Bourgogne Franche-Comté, 2500 Besançon, France; 5 Department of Movement and Sports Sciences, Ghent University, 9000 Ghent, Belgium; 6 Division of Health, Engineering, Computing and Science, Te Huataki Waiora School of Health, Adams Centre for High Performance, University of Waikato, Tauranga, 3116 New Zealand; 7 Department of Sports Science, National Sports Institute of Malaysia, 57000 Kuala Lumpur, Malaysia

**Keywords:** Bone quality and biomechanics, Population screening

## Abstract

Running biomechanics and ethnicity can influence running economy (RE), which is a critical factor of running performance. Our aim was to compare RE of South East Asian (SEA) and non-South East Asian (non-SEA) runners at several endurance running speeds (10–14 km/h) matched for on-road racing performance and sex. Secondly, we explored anthropometric characteristics and relationships between RE and anthropometric and biomechanical variables. SEA were 6% less economical (*p* = 0.04) than non-SEA. SEA were lighter and shorter than non-SEA, and had lower body mass indexes and leg lengths (*p* ≤ 0.01). In terms of biomechanics, a higher prevalence of forefoot strikers in SEA than non-SEA was seen at each speed tested (*p* ≤ 0.04). Furthermore, SEA had a significantly higher step frequency (*p* = 0.02), shorter contact time (*p* = 0.04), smaller footstrike angle (*p* < 0.001), and less knee extension at toe-off (*p* = 0.03) than non-SEA. Amongst these variables, only mass was positively correlated to RE for both SEA (12 km/h) and non-SEA (all speeds); step frequency, negatively correlated to RE for both SEA (10 km/h) and non-SEA (12 km/h); and contact time, positively correlated to RE for SEA (12 km/h). Despite the observed anthropometric and biomechanical differences between cohorts, these data were limited in underpinning the observed RE differences at a group level. This exploratory study provides preliminary indications of potential differences between SEA and non-SEA runners warranting further consideration. Altogether, these findings suggest caution when generalizing from non-SEA running studies to SEA runners.

## Introduction

Running economy (RE), which refers to steady-state oxygen consumption at a given submaximal running speed, is a critical factor of running performance^1^. RE has been shown to differ between ethnic groups^2–5^. Indeed, Weston, et al.^2^ noted greater RE in African than Caucasian distance runners though not elucidating the origin of these differences. Similarly, elite Kenyans were found more economical than their Caucasian counterparts^3–5^. This difference was attributed to body dimensions, with longer legs (~ 5%), thinner and lighter calf musculature, as well as lower body mass and body mass index (BMI) in Kenyans than Caucasians, but not to differences in muscle fibre type^3–6^. These findings may partially explain the success of African runners at the elite level. Indeed, the longer, slenderer legs of Kenyans could be advantageous when running as RE is correlated with leg mass^6^. However, the precise mechanisms underpinning anthropometric and economy relationships are not clear^7^.

Research into running and ethnic differences has mostly compared Caucasian and African runners^2–5,8–13^. These studies highlight differences in physiological^2–5,12^, anthropometrical^9,14^, neuromuscular^15^, and running gait patterns^8,10,11^ between ethnicities. Altogether, these results indicate caution in the generalization of results from one ethnic group to another.

There exists only limited inclusion of Asian cohorts in running studies^14–16^ and, to the best of our knowledge, no study comparing their RE to another ethnic group. Nonetheless, road race participation continues to grow in Asia despite a decline in the number of participants since 2016 outside of Asia^17^. Therefore, the relative underrepresentation of Asian runners in research is of concern, especially when considering their unique anthropometric features^18,19^, autonomic responses to exercise^20^, muscle–tendon unit properties^15^, walking gait characteristics^21^, and footstrike patterns^16^ compared to other ethnic groups.

Although running biomechanics can influence RE^1^, the relationships between select biomechanical variables and RE are unclear and even conflicting in the scientific literature. For instance, Gruber, et al.^22^ observed no difference in RE between rearfoot (RFS) and non-rearfoot (non-RFS) strike patterns, while both RFS^23^ and non-RFS^24^ patterns were suggested as more economical than the other. Similarly, superior RE has been linked with both long^25^ and short^26^ ground contact times (*t*_*c*_), while Williams and Cavanagh^27^ found no significant relation between RE and *t*_*c*_. These divergent findings might be due to differences between the cohorts examined, including ethnic differences.

For these reasons, our primary aim was to explore whether South East Asian (SEA) and non-South East Asian (non-SEA) runners demonstrate similar RE at several endurance running speeds when matched for on-road running performance and sex. Secondly, we aimed to explore anthropometric differences between groups and potential relationships between RE and anthropometric and biomechanical variables in these groups.

## Materials and methods

### Participants

An existing database of 54 runners was explored to match SEA and non-SEA runners based on sex and on-road running performance on 21.1 km^28^. The matching led to the inclusion of 34 trained runners, 20 males (variable: mean ± standard deviation, age: 36 ± 6 years, mass: 68 ± 11 kg, height: 176 ± 7 cm, leg length: 92 ± 5 cm, BMI: 22 ± 2 kg/m^2^, running distance: 56 ± 20 km/week, running experience: 9 ± 7 y, and best half-marathon time: 93 ± 9 min) and 14 females (age: 36 ± 6 y, mass: 53 ± 6 kg, height: 162 ± 4 cm, leg length: 84 ± 3 cm, BMI: 20 ± 2 kg/m^2^, running distance: 58 ± 17 km/week, running experience: 7 ± 5 years, and best half-marathon time: 100 ± 9 min) in this study. For study inclusion, participants were required to be in good self-reported general health with no current or recent (< 3 months) musculoskeletal injuries and to meet a certain level of running performance. More specifically, runners were required to have competed in a road race in the last year with finishing times of ≤ 50 min for 10 km, ≤ 1 h 50 min for 21.1 km or ≤ 3 h 50 min for 42.2 km. The ethical committee of the National Sports Institute of Malaysia approved the study protocol prior to participant recruitment (ISNRP: 26/2015), which was conducted in accordance with international ethical standards^29^ and adhered to the latest Declaration of Helsinki of the World Medical Association.

Runners were classified in two ethnic groups based on their nationality: SEA and non-SEA, which led to a total of 17 participants per group. SEA runners were from China (*n* = 12), Malaysia (*n* = 14), and Indonesia (*n* = 1); while non-SEA runners were from England (*n* = 7), Sweden (*n* = 2), Australia, Brazil, Canada, Denmark, France, Norway, Poland, and Scotland (*n* = 1 each). All non-SEA runners identified as “white”.

### Experimental procedure

Each participant completed one experimental laboratory session. After providing written informed consent, the right leg length of participants was measured (from anterior superior iliac spine to medial malleolus in supine). Participants then ran 5 min at 9 km/h on a treadmill (h/p/cosmos mercury®, h/p/cosmos sports & medical gmbh, Nussdorf-Traunstein, Germany) as a warm-up. Participants then completed 3 × 4-min runs at 10, 12, and 14 km/h (with 2-min recovery periods between runs) on the treadmill, during which time RE was assessed. Retro-reflective markers were subsequently positioned on individuals (described in *Data Collection* section) to assess running kinematics. For each participant, a 1-s static calibration trial was recorded, which was followed by 3 × 30-s runs at 10, 12, and 14 km/h (with 1-min recovery periods between each runs) to collect three-dimensional (3D) kinematic data in the last 10-s segment of these runs (30 ± 2 running steps), resulting in at least 25 steps being analysed^30^. RE and biomechanics were assessed separately given laboratory constraints and interference with data quality (e.g., presence of testing equipment that occluded markers). All participants were familiar with running on a treadmill as part of their usual training programs and wore their habitual running shoes during testing.

### Data collection

Gas exchange was measured using TrueOne 2400 (ParvoMedics, Sandy, UT, USA) during the 3 × 4-min runs. Prior to the experiment, the gas analyzer was calibrated using ambient air (O_2_: 20.93% and CO_2_: 0.03%) and a gas mixture of known concentration (O_2_: 16.00% and CO_2_: 4.001%). Volume calibration was performed at different flow rates with a 3 L calibration syringe (5530 series, Hans Rudolph, Shawnee, KS, USA). Oxygen consumption (${\dot{\text{V}}\text{O}}_{2}$), carbon dioxide production (${\dot{\text{V}}\text{CO}}_{2}$), and respiratory exchange ratio (RER) values were averaged over the last minute of each 4-min run. Steady state was confirmed through visual inspection of the ${\dot{\text{V}}\text{O}}_{2}$ and ${\dot{\text{V}}\text{CO}}_{2}$ curves for all running trials. RER had to remain below unity during the trials for data to be included in the analysis, otherwise the corresponding data were excluded as deemed to not represent a submaximal effort. No trial was excluded on this basis. RE was expressed as the oxygen cost per mass to the power of 0.75 per kilometer (ml/kg^0.75^/km) to minimize the influence of body mass per se on ${\dot{\text{V}}\text{O}}_{2}$ during running^31^. RE expressed in ml/kg/km was also computed for reference and is provided as supplementary materials. A higher RE value indicates a less economical runner.

**Figure 1:**
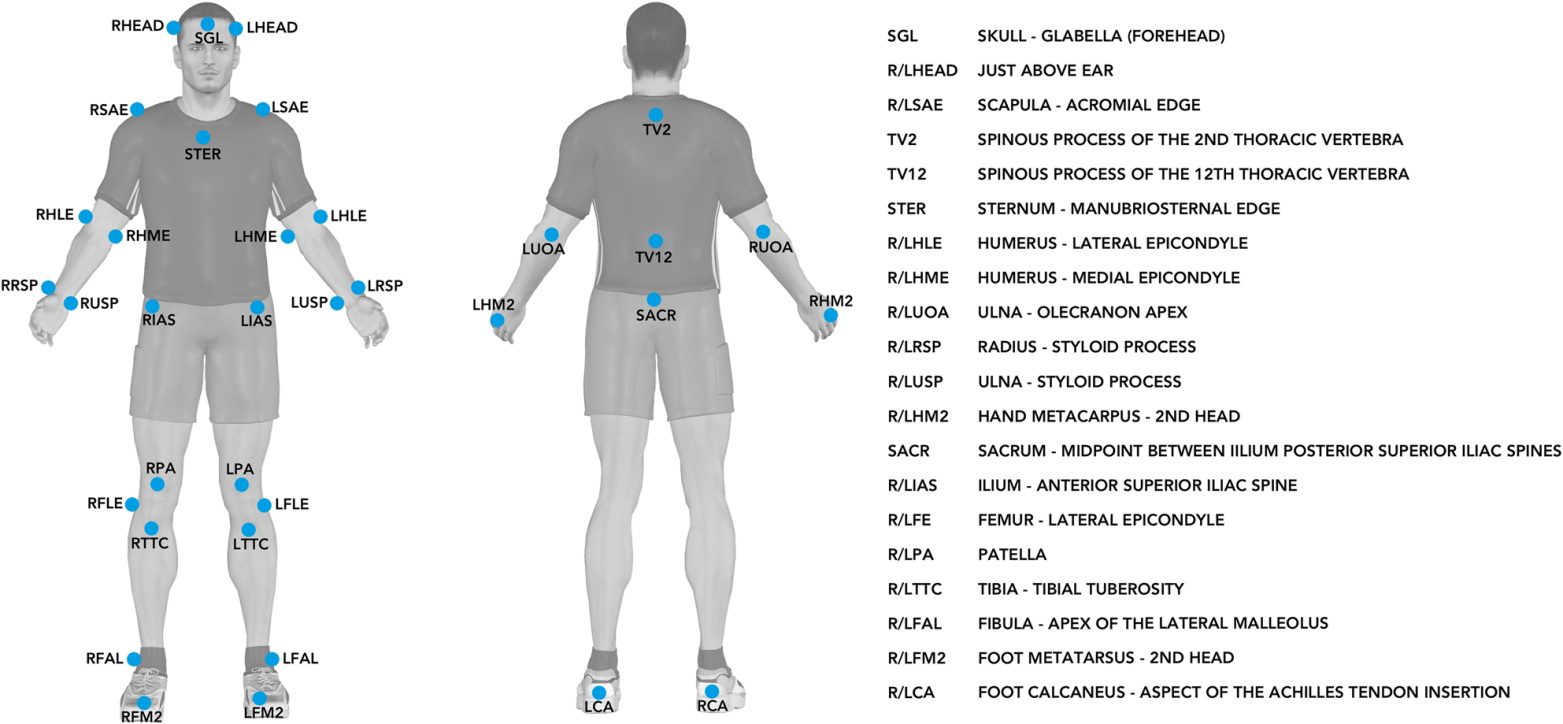
Retro-reflective markers (*N* = 35) placed on anatomical landmarks of participants for biomechanical data collection. R and L at the start of the acronyms denote right and left, respectively.

3D kinematic data were collected at 200 Hz using seven infrared Oqus cameras (five Oqus 300+, one Oqus 310+, and one Oqus 311+) and Qualisys Track Manager software version 2.1.1 build 2902 together with the Project Automation Framework Running package version 4.4 (Qualisys AB, Göteborg, Sweden). A virtual laboratory coordinate system was generated such that the *x*–*y*–*z* axes denoted the medio-lateral (pointing towards the right side of the body), posterior-anterior, and inferior-superior directions, respectively. Thirty-five retro-reflective markers (Fig. 1) of 12 mm in diameter were used for static calibration and running trials, and were affixed to the skin and shoes of individuals over anatomical landmarks using double-sided tape following standard guidelines from the Project Automation Framework Running package^32^. The 3D marker data were exported in .c3d format and processed in Visual3D Professional software version 5.02.25 (C-Motion Inc., Germantown, MD, USA). More explicitly, the 3D marker data were interpolated using a third-order polynomial least-square fit algorithm, allowing a maximum of 20 frames for gap filling, and subsequently low-pass filtered at 20 Hz using a fourth-order Butterworth filter.

### Biomechanical variables

From the marker set, a full-body biomechanical model with six degrees of freedom at each joint and 15 rigid segments was constructed. The model included the head, upper arms, lower arms, hands, thorax, pelvis, thighs, shanks, and feet. Segments were assigned inertial properties and centre of mass (COM) locations based on their shape^33^ and attributed relative mass based on standard regression equations^34^. Kinematic variables were calculated using rigid-body analysis and whole-body COM location was calculated from the parameters of all 15 segments. Ankle ($\theta_{{{\text{ankle}}}}$) and knee ($\theta_{{{\text{knee}}}}$) joint angles were defined as the orientation of the distal segment relative to the proximal one^35^. Angles were computed using an *x–y–z* Cardan sequence^36,37^ equivalent to the joint coordinate system^36,38^, leading to rotations with functional and anatomical meaning (flexion–extension, abduction–adduction, and internal–external rotation). Noteworthy, only the flexion–extension Cardan angle was considered for analysis due to possible errors linked with kinematic crosstalk^39–41^. Joint angles were calculated at footstrike and toe-off events. Footstrike angle (FSA) was calculated following the procedure described in Altman and Davis^42^. FSA was normalized by taking the angle of the foot at footstrike and subtracting the angle of the foot during standing trial. The mean FSA was used to categorise footstrike patterns of runners in two categories: RFS when the FSA was greater than 8°, and non-RFS when 8° or less^42^. Among all running trials, 5% and 7% were borderline (within 1°) RFS and non-RFS, respectively. These borderline footstrike patterns were only present in SEA runners. Visual inspection confirmed the footstrike pattern classifications were correct.

Running events were derived from the trajectories of the 3D marker data using similar procedures to those previously reported ^43,44^. All events were verified to ensure correct identification and were manually adjusted when required.

Swing time (*t*_*s*_) and *t*_*c*_ were defined as the time from toe-off to footstrike and from footstrike to toe-off of the same foot, respectively. Flight time (*t*_*f*_) was defined as the time from toe-off to footstrike of the contralateral foot. Step frequency (SF) was calculated as ${\text{SF}} = \frac{1}{{t_{c} + t_{f} }}$, and step length (SL) as ${\text{SL}} = s/{\text{SF}}$, where *s* represents running speed. In addition to raw units, SL was expressed as a percentage of participant's leg length. The spring-mass characteristics of the lower limb were estimated using a sine-wave model following the procedure defined by Morin, et al.^45^. More explicitly, leg stiffness (*k*_leg_) was calculated as [Eq. (1)]

1 $$k_{{{\text{leg}}}} = \frac{{F_{{z,{\text{max}}}} }}{\Delta L}$$

where *F*_*z,*max_ represents the maximal vertical force and was estimated using $F_{{z,{\text{max}}}} = mg\frac{\pi }{2}\left( {\frac{{t_{f} }}{{t_{c} }} + 1} \right)$, $\Delta L$ is the maximal leg length deformation, i.e., the leg spring compression and given by $\Delta L = \sqrt {z_{{{\text{COM}},{\text{FS}}}}^{2} + s^{2} t_{b}^{2} } - z_{{{\text{COM}},{\text{MS}}}}$, where *s* defines running speed, *t*_*b*_ denotes the braking time, i.e., the time from footstrike to mid-stance, and z_COM,FS_ and z_COM,MS_ are the COM heights at footstrike and mid-stance, respectively. For all biomechanical measures, the values extracted from the 10-s data collection for each participant were averaged for subsequent statistical analyses.

### Statistical analysis

Descriptive statistics are presented using mean ± standard deviation (SD). Data normality and homogeneity of variances were verified using Kolmogorov–Smirnov and Levene's test, respectively. Participant characteristics between SEA and non-SEA runners were compared using unpaired two-sided Welch's *t*-tests when homogeneity of variance assumptions were violated and unpaired two-sided Student's *t*-tests otherwise. The effect of group (SEA, non-SEA) and running speed on RE and biomechanical variables was evaluated using a linear mixed effects model fitted by restricted maximum likelihood. The within-subject nature was controlled for by including random effects for participants (individual differences in the intercept of the model). The fixed effects included group and running speed (both categorical variables). Cohen's *d* effect size was calculated when a significant group effect was observed^46^, and classified as *small*, *moderate*, and *large* when *d* values were larger than 0.2, 0.5, and 0.8, respectively^46^. Footstrike distribution between SEA and non-SEA runners were compared at all running speeds using Fisher exact tests given that some of the expected frequencies were less than five.

A correlation matrix between anthropometric characteristics (mass and height, leg length, BMI, and ratio of leg length over height) was generated to identify unrelated anthropometric characteristics. Pearson correlation coefficients (*r*) between RE and the identified independent anthropometric variables were computed using RE values at the three running speeds separately, as well as with and without subgrouping of participants based on ethnicity. Similarly, Pearson correlation coefficients (*r*) between RE and biomechanical variables were computed at the three running speeds separately, as well as with and without subgrouping of participants based on ethnicity. Correlations were considered *very high*, *high*, *moderate*, *low*, and *negligible* when absolute *r* values were between 0.90–1.00, 0.70–0.89, 0.50–0.69, 0.30–0.49, and 0.00–0.29, respectively^47^. Given the number of correlations and exploratory nature of these analyses, only significant correlations reaching the *moderate* threshold were deemed meaningful. Statistical analyses were performed using Jamovi (version 1.2.17, Computer Software, retrieved from https://www.jamovi.org) and R (version 3.5.0, The R Foundation for Statistical Computing, Vienna, Austria) with a level of significance set at *p* ≤ 0.05.

## Results

### Participant characteristics

Non-SEA runners were significantly heavier and taller, had a larger BMI and longer legs, had footwear with a larger heel-to-toe drop, and were more experienced than SEA runners (*p* ≤ 0.02; Table 1). Otherwise, demographic and footwear characteristics of non-SEA and SEA runners were similar (see Table 1).

**Table 1:** Participant and footwear characteristics for South East Asian (SEA) and non-South East Asian (non-SEA) runners.

Characteristics	SEA	Non-SEA	*p*
Sex	M = 10; F = 7	M = 10; F = 7	NA
Age (y)	34 ± 4	38 ± 7	0.08
Mass (kg)	56 ± 9	68 ± 12	**0.002**
Height (cm)	167 ± 8	175 ± 9	**0.01**
Leg length (cm)	86 ± 4	91 ± 6	**0.01**
BMI (kg/m^2^)	20 ± 2	22 ± 2	**0.004**
Leg length over height (%)	52 ± 1	52 ± 1	0.54
Running distance (km/week)	60 ± 19	54 ± 18	0.32
Running experience (y)	6 ± 3	11 ± 7	**0.02**
Running performance on 21.1 km (min)	96 ± 9	96 ± 10	0.81
Shoe mass (g)	231 ± 32	215 ± 39	0.22
Shoe stack height (mm)	25 ± 3	25 ± 3	0.83
Shoe heel-to-toe drop (mm)	8 ± 3	6 ± 3	**0.01**

Significant differences (*p* ≤ 0.05) identified by Student's or Welch's t-tests are reported in bold.

*M* male, *F* female, *BMI* body mass index, and *NA* not applicable.

### Running economy

**Figure 2:**
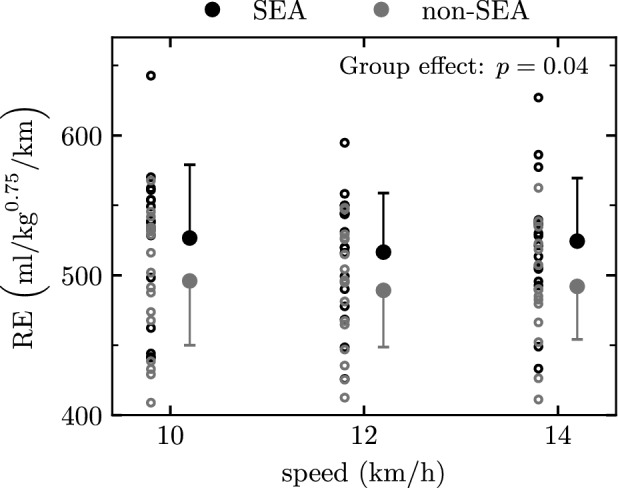
Running Economy (RE) of South East Asian (SEA) and non-South East Asian (non-SEA) runners at several endurance running speeds. Linear mixed effects modelling identified a significant group effect (*p* ≤ 0.05).

SEA runners were significantly less economical (6%) than non-SEA runners (average across speeds: 522.6 ± 47.4 vs 492.4 ± 42.2 ml/kg^0.75^/km), with a *moderate* main effect of group on RE (*p* = 0.04, *d* = 0.67; Fig. 2). There was no significant main effect of speed (*p* = 0.27) or group x speed interaction effect (*p* = 0.89) on RE. Larger differences were seen between SEA and non-SEA runners when expressing RE in ml/kg/km instead of ml/kg^0.75^/km (see section S1 of supplementary materials).

### Biomechanical characteristics

There was a significant main effect of group on SF, SL, and *t*_*c*_ (*p* ≤ 0.04; Table 2), with SEA having a higher SF (*moderate* effect; *d* = 0.75), smaller SL (*small* effect; *d* = 0.36), and shorter *t*_*c*_ (*moderate* effect; *d* = 0.67) than non-SEA runners. There was no group effect on normalized SL, *t*_*f*_ and *k*_leg_ (*p* ≥ 0.23; Table 2). A significant speed effect was observed for all temporal variables (*p* ≤ 0.01; Table 2). SF, SL, and *t*_*f*_ increased with increasing speed, whereas *t*_*c*_ and *k*_leg_ decreased with increasing speed. None of these variables demonstrated a group x speed interaction (*p* ≥ 0.32; Table 2).

**Table 2:** Step frequency (SF), step length (SL), contact time (*t*_*c*_), flight time (*t*_*f*_), and spring-mass characteristics of the lower limb as given by leg stiffness (*k*_leg_) for South East Asian (SEA) and non-South East Asian (non-SEA) runners at endurance running speeds.

Running speed (km/h)	Group	SF (steps/min)	SL (cm)	SL (%)^a^	*t*_*c*_ (ms)	*t*_*f*_ (ms)	*k*_leg_ (kN/m)
10	SEA	176 ± 9	95 ± 5	110 ± 7	268 ± 24	78 + 21	12.3 ± 2.5
	Non-SEA	168 ± 9	100 ± 5	110 ± 6	287 ± 31	84 + 23	13.5 ± 2.8
12	SEA	181 ± 10	111 ± 6	128 ± 8	237 ± 22	96 ± 21	12.3 ± 2.4
	Non-SEA	173 ± 10	116 ± 7	127 ± 6	253 ± 23	98 ± 25	13.5 ± 3.0
14	SEA	187 ± 11	125 ± 7	145 ± 9	215 ± 20	107 ± 19	12.0 ± 2.2
	Non-SEA	179 ± 11	131 ± 8	144 ± 7	231 ± 21	107 ± 23	12.8 ± 2.7
Group effect		**0.02**	**0.03**	0.78	**0.04**	0.67	0.23
Running speed effect		** < 0.001**	** < 0.001**	** < 0.001**	** < 0.001**	** < 0.001**	**0.009**
Interaction effect		0.93	0.48	0.68	0.81	0.44	0.32

Significant differences (*p* ≤ 0.05) identified by linear mixed effects modelling are indicated in bold. SL was expressed as a percentage of participant's leg length in addition to raw units.

^a^Step length normalized to leg length.

There was a significant group effect on $\theta_{{{\text{ankle}}}}$ at footstrike and $\theta_{{{\text{knee}}}}$ at toe-off (*p* ≤ 0.03; Table 3), with SEA having less ankle dorsiflexion than non-SEA at footstrike (large effect; d = 1.20) and less knee extension at toe-off (moderate effect; d = 0.75). A significant speed effect was observed for $\theta_{{{\text{ankle}}}}$ and $\theta_{{{\text{knee}}}}$ at toe-off (*p* ≤ 0.02; Table 3), with greater flexion at footstrike and extension at toe-off with increasing speed. None of these variables showed a group x speed interaction except $\theta_{{{\text{ankle}}}}$ at footstrike (*p* = 0.007; Table 3), with SEA decreasing dorsiflexion with increasing speed while non-SEA increased dorsiflexion with increasing speed.

**Table 3:** Flexion–extension angle of the lower limb for South East Asian (SEA) and non-South East Asian (non-SEA) runners at endurance running speeds.

Running speed (km/h)	Group	$\theta_{{{\text{ankle}}}}$(°)		$\theta_{{{\text{knee}}}}$(°)	
		FS	TO	FS	TO
10	SEA	9 ± 5	− 12 ± 8	17 ± 2	27 ± 4
	Non-SEA	14 ± 6	− 9 ± 3	18 ± 3	24 ± 7
12	SEA	8 ± 5	− 14 ± 8	17 ± 3	24 ± 4
	Non-SEA	15 ± 6	− 11 ± 3	18 ± 4	21 ± 5
14	SEA	8 ± 6	− 14 ± 9	18 ± 3	24 ± 4
	Non-SEA	15 ± 6	− 11 ± 4	18 ± 4	20 ± 4
Group effect		**0.001**	0.18	0.57	**0.03**
Running speed effect		0.31	**0.02**	0.65	** < 0.001**
Interaction effect		**0.007**	0.95	0.09	0.65

Significant differences (*p* ≤ 0.05) identified by linear mixed effects modelling are indicated in bold.

$\theta_{{{\text{ankle}}}}$: ankle joint angle, $\theta_{{{\text{knee}}}}$: knee joint angle, FS: footstrike, and TO: toe-off.

### Footstrike angle and pattern

SEA had a significantly lower FSA than non-SEA runners (*large* effect; *d* = 1.67), as depicted by the group effect on FSA (*p* < 0.001; Table 4). A speed effect was observed on FSA (*p* < 0.001; Table 4), indicating an increase of FSA with increasing running speed, while no significant group x speed interaction effect was noted (*p* = 0.13; Table 4). Footstrike distribution between SEA and non-SEA runners differed significantly at all speeds, with non-SEA being more commonly RFS (*p* ≤ 0.04; Table 4).

**Table 4:** Footstrike angle (FSA) and footstrike distribution [rearfoot strike (RFS) for FSA > 8° and non-rearfoot strike (non-RFS) otherwise^42^] for South East Asian (SEA) and non-South East Asian (non-SEA) runners at endurance running speeds.

Running speed (km/h)	Group	FSA (°)	RFS—non-RFS	*p*
10	SEA	6 ± 4	4–13	** < 0.001**
	Non-SEA	13 ± 5	16–1	
12	SEA	7 ± 4	6–11	** < 0.001**
	Non-SEA	15 ± 5	16–1	
14	SEA	9 ± 4	10–7	**0.04**
	Non-SEA	17 ± 6	16–1	
Group effect		** < 0.001**	NA	
Running speed effect		** < 0.001**	NA	
Interaction effect		0.13	NA	

Significant differences (*p* ≤ 0.05) identified by linear mixed effects modelling and by Fisher exact tests are indicated in bold.

### Relationship between RE and anthropometric characteristics

*High* positive correlations were identified between mass and height (*r* ≥ 0.83; *p* < 0.001), mass and leg length (*r* ≥ 0.74; *p* < 0.001), and mass and BMI (*r* ≥ 0.84; *p* < 0.001), while the correlation between mass and ratio of leg length over height was negligible and not significant (*r* ≤ 0.17; *p* ≥ 0.35). Hence, relationships between RE and mass and ratio of leg length over height were further explored (Table 5). For SEA runners, a *high* positive correlation was observed between RE and mass at 12 km/h (*r* = 0.69, *p* < 0.001; Table 5), while *high* positive correlations were observed between RE and mass for non-SEA runners at all speeds (*r* ≥ 0.65, *p* ≤ 0.005; Table 5). For runners combined, the strongest correlations were *low.* Table 6 presents all correlations, including the *low* and *negligible* ones. Relationships between RE expressed in ml/kg/km and anthropometric characteristics are provided in section S1 of supplementary materials.

**Table 5:**
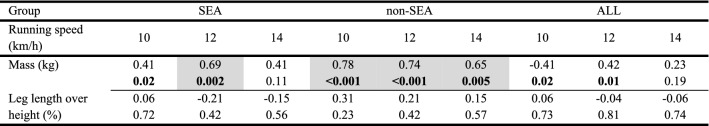
Pearson correlation coefficients between running economy and anthropometric characteristics (mass and ratio of leg length over height), together with their corresponding *p*-values underneath for South East Asian (SEA), non-South East Asian (non-SEA), as well as all runners pooled together (ALL).

*Note.* Only the relationships between running economy and mass and ratio of leg length over height were considered because mass was highly and significantly correlated to height, leg length, and body mass index.

Statistical significances (*p* ≤ 0.05) gray shaded boxes denote correlation coefficients above an absolute value of 0.5 (moderate).

**Table 6:**
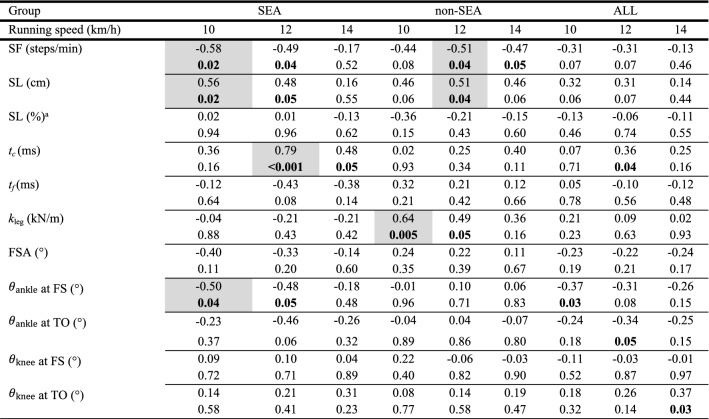
Pearson correlation coefficients between running economy and biomechanical variables [step frequency (SF), step length (SL), contact time (t_c_), flight time (t_f_), spring-mass characteristics of the lower limb as given by leg stiffness (k_leg_), footstrike angle (FSA), and flexion–extension ankle ($\theta_{{{\text{ankle}}}}$) and knee ($\theta_{{{\text{knee}}}}$) joint angle at footstrike (FS) and toe-off (TO)], together with their corresponding *p*-values underneath for South East Asian (SEA), non-South East Asian (non-SEA), as well as all runners pooled together (ALL).

Statistical significances (*p* ≤ 0.05) are indicated in bold. Gray shaded boxes denote correlation coefficients above an absolute value of 0.5 (moderate). SL was expressed as a percentage of participant's leg length in addition to raw units.

^a^Step length normalized to leg length.

### Relationships between RE and biomechanics

For SEA runners, a *high* positive correlation was seen between RE and *t*_*c*_ at 12 km/h (|*r*|≥ 0.70, *p* ≤ 0.002; Table 6). SF and $\theta_{{{\text{ankle}}}}$ at footstrike at 10 km/h were *moderately* and negatively correlated to RE, whereas SL (10 km/h) was *moderately* and positively correlated to RE (|*r*|≥ 0.50, *p* ≤ 0.04; Table 6).

For non-SEA runners, a *moderate* and negative correlation was observed between RE and SF at 12 km/h (|*r*|≥ 0.51, *p* ≤ 0.04; Table 6). Besides, *moderate* positive correlations between RE and SL (12 km/h) and *k*_leg_ (10 km/h) were identified (|*r*|≥ 0.51, *p* ≤ 0.04; Table 6).

For runners combined, the strongest correlations were *low.* Table 6 presents all correlations, including the *low* and *negligible* ones. Relationships between RE expressed in ml/kg/km and biomechanics are given in section S1 of supplementary materials.

## Discussion

Differences in RE were observed between SEA and non-SEA runners despite being matched for recent (< 1 year) road running performance and sex. SEA runners were less economical than non-SEA runners at endurance running speeds. Anthropometric differences were observed between groups, depicting that SEA were lighter and shorter than non-SEA runners, and had a lower BMI and shorter legs. Differences in running biomechanics between cohorts were also observed, but correlations between anthropometric and biomechanical variables and RE measures at a group-level were of *small* magnitudes at best, and provided limited explanations of the underlying differences in RE.

Non-SEA were 6% more economical than SEA runners at endurance running speeds (Fig. 2). The lower RE in SEA than non-SEA runners could in part be due to anthropometric differences. We observed that SEA were lighter and shorter than non-SEA runners, and had a lower BMI and shorter legs (Table 1). Mass was significantly related to RE in both ethnic groups, with more economical runners having lower body mass. Mass was highly related to RE in SEA runners at 12 km/h and non-SEA at all speeds, but correlations became *low* or non-significant when pooling all runners together (Table 5). Previous studies showing that elite Caucasian runners were less economical than Kenyans attributed RE differences to longer legs (~ 5%), thinner and lighter calf musculature, and lower mass and BMI of Kenyan than Caucasian runners^3–6^. Indeed, RE being correlated with leg mass, Kenyan runners could benefit from their long, slender legs^6^. In our case, the ratio of leg length over height was not related to RE (Table 5) and was similar between SEA and non-SEA, indicating similar lower limb proportions in these two groups (Table 1). In fact, due to both smaller mass and shorter legs (Table 1), SEA might have had a proportionally similar leg mass than non-SEA runners.

Participants wore their own running shoes during testing similar to previous research exploring differences in running mechanics between ethnic groups^10^. Given that differences in footwear characteristics can underpin differences in running biomechanics^48^ and RE^49^, using a standardised shoe might have led to different study outcomes. Noteworthy, however, is that there were no significant difference in shoe mass or stack height between groups, with the 2 mm difference in heel-to-toe drop between groups likely having limited biomechanical or performance implications^50^. Recreational runners are more comfortable wearing their own shoes^51^, and show individual responses to novel footwear^51,52^ and cushioning properties^53^. A recent meta-analysis indicates recreational runners demonstrate improved RE when wearing more comfortable shoes^54^, supporting the appropriateness of participants wearing their own footwear for this investigation. Nevertheless, it is possible that other footwear characteristics not assessed as part of this study differed between groups, such as midsole cushioning and/or the longitudinal bending stiffness^50^, and contributed to the biomechanical and RE differences observed.

Among all correlations between biomechanical variables and RE, only SF and SL were significantly related to RE in both ethnic groups. The SF and SL variables were moderately related to RE in SEA runners at 10 km/h and non-SEA at 12 km/h, but correlations became *low* and non-significant when all runners were pooled together (Table 6). Noteworthy, correlations between SL and RE were smaller and became non-significant when normalized to leg length. In addition, *t*_*c*_ was highly and positively related to RE for SEA runners at 12 km/h. The identified correlations between SF (and SL) and RE and between *t*_*c*_ and RE suggest that individuals with higher SF (and shorter SL) and smaller *t*_*c*_ (for SEA runners) are more economical. However, SEA had intrinsically higher SF (and shorter SL) and shorter *t*_*c*_, but worse RE than non-SEA runners (Table 2); therefore, contradicting the observed correlations. Based on the cost-of-generating-force hypothesis, one requires less metabolic energy with increased *t*_*c*_ and longer leg lengths^55–57^, both observed in non-SEA (Table 1). The longer *t*_*c*_ in non-SEA suggests that muscles had more time to shorten and produce the necessary forces to move the body than SEA runners. Based on the force–velocity relationship, if a muscle is shortening slower but only a given force is necessary (i.e., running on a treadmill), it could be speculated that the activation levels of the muscles were lower to reach the target force. These theories might partially explain the reduced metabolic cost in non-SEA than SEA runners, i.e., a longer *t*_*c*_, lower SF, and longer leg lengths are more economical.

Nevertheless, studies indicate that increasing SF above self-selected ones in novice (156 ± 6 steps/min, 9.6 km/h) and trained (169 ± 11 steps/min, 12.6 km/h) runners acutely improves RE (+ 2%)^58^, as does undertaking a 10-day training programme to increase SF (from 166 ± 4 to 180 ± 1 steps/min, 12.3 km/h)^59^. At 12 km/h, mean SF values were 173 (range: 151 to 185) in non-SEA and 181 (range: 159 to 200) in SEA. Further increasing SF in runners with an intrinsically high SF might not be energetically optimal, but has yet to be examined. An extremely high SF might be suboptimal at endurance speeds given the greater mechanical power associated with increased frequency of reciprocal movements, which may require a greater reliance on less economical type II muscle fibers^60^. Indeed, Kaneko et al.^60^ suggested that SF and RE could be related through muscle fiber recruitment. Besides, given the shorter stature of our SEA vs non-SEA runners, their higher SF aligns with findings of moderate correlations between leg lengths and SF (*r* = −0.53, *p* < 0.001; 12 km/h), in agreement with previous literature (*r* = −0.45, *p* < 0.001) ^61^, whereby individuals with shorter legs tend to adopt higher SF.

Alongside their higher SF and smaller SL, SEA had shorter *t*_*c*_, smaller FSA (more forefoot strike pattern), and smaller ${ }\theta_{{{\text{ankle}}}}$ at footstrike than non-SEA runners (Tables 2, 3, 4). Previous studies observed that running at a higher SF led to smaller *t*_*c*_^62^ and FSA^63^, which is consistent with our findings. In addition, the prevalence of RFS was shown to be lower in Asian than North American recreational runners^16^, aligning with the findings of the present study. A smaller *t*_*c*_ might be associated with smaller braking and propulsion phases. Although short braking phases are considered important for economical running^64^, SEA runners were less economical. Braking forces were not recorded herein due to unavailability of instrumented treadmills. Shorter braking times does not necessarily equate minimising braking forces, which is important in the context of RE^65^. Moreover, it could be that the orientation of the ground reaction forces in SEA runners was suboptimal. Indeed, Moore, et al.^66^ observed that a better alignment of the leg axis during propulsion and resultant ground reaction force improved RE, mainly via a more horizontal application of the ground reaction force. This idea is supported by our data, which show less extension of $\theta_{{{\text{knee}}}}$ at toe-off (Table 3), and thus potentially less horizontal propulsion for SEA than non-SEA runners. Nevertheless, $\theta_{{{\text{knee}}}}$ at toe-off was not correlated to RE (Table 6). Though SF, SL and *t*_*c*_ significantly differed between groups, no difference in *k*_leg_ was identified (Table 2), contradicting previous findings that *k*_leg_ relates to the aforementioned variables^67–69^. These studies were all within-subject comparisons rather than between-subject ones; hence, at an individual level, the relationship might still hold within SEA and non-SEA participants. The lack of difference in *k*_leg_ between groups despite differences in SF, SL, and *t*_*c*_ potentially relates to the body mass difference between groups that is counterbalancing the spatiotemporal differences in the biomechanical variables [see Eq. (1)]. These biomechanical data were not clearly able to explain the variances in RE between groups, and support that RE improvements in various groups might need individualized training and considerations. A similar conclusion was made by Santos-Concejero, et al.^10^ when assessing RE differences between Eritrean and European runners. Moreover, these divergent findings overall suggest there is no unique or ideal running pattern that is the most economical amongst runners^1^. The running pattern of an individual results from a complex interaction between several biomechanical factors ^70^ that are interconnected and interact in a global and dynamic manner^71^ to optimize RE.

A few limitations to the present study exist. Although the effect size was *moderate* (*d* = 0.67), the between-group difference in RE units was rather small (mean difference = 30.1 ml/kg^0.75^/km; *p* = 0.04). In addition, the within-group variability in RE and biomechanical variables at a given running speed were relatively small. Therefore, observed correlations between RE and biomechanical variables might have been greater in more heterogeneous groups. Given the exploratory nature of this investigation, several variables were compared, leading to a high likelihood of finding a spurious difference or correlation. Nonetheless, our research provides preliminary indications of potential differences between SEA and non-SEA runners warranting further consideration. Moreover, an underpinning factor to the differences in RE might be the running experience given that experienced runners self-optimize their running patterns better than novice runners^1^. Non-SEA runners were more experienced (years running) than SEA runners (Table 1), but all runners trained regularly and had a minimum of 2 years running experience, indicating they were all "experienced" and not "novice" runners. Nonetheless, a gradual improvement in RE (+ 15%) over an 11-year time span has been reported for a former world record holder in the women’s marathon^72^. Therefore, an effect due to running experience cannot be ruled out. Besides, several morphological factors which were not measured in this study might have partly explained differences in RE between SEA and non-SEA runners^18,19,73–78^ (more details are provided in section S2 of supplementary materials). Furthermore, although all SEA runners identified as “white”, the numerous nationalities of the non-SEA group potentially increased the heterogeneity of our cohort and influenced our results. Lastly, RE and biomechanics were collected within the same experimental session, but the two were not collected simultaneously (as common in running research^79^). Although possible that participants altered their runs, research indicates that metabolic equipment does not affect sagittal plane running kinematics and are comparable to running without metabolic testing^80^.

## Conclusion

SEA and non-SEA runners were different in terms of RE, with SEA runners being less economical than non-SEA runners at endurance running speeds. Differences in anthropometric characteristics and running biomechanics between cohorts were also observed, but explained differences in RE to a limited extent. Other factors, which could be related to ethnicity, might be underpinning such differences. Unfortunately, these factors were not measured in this study. Nonetheless, caution must be taken when generalizing from non-SEA running studies to SEA runners.

## Supplementary Information

Supplementary Information.

## Data Availability

The dataset supporting this article is available on request to the corresponding author.

## References

[1] Moore IS (2016). Is there an economical tunning technique? A review of modifiable biomechanical factors affecting running economy. Sports Med..

[2] Weston AR, Mbambo Z, Myburgh KH (2000). Running economy of African and Caucasian distance runners. Med. Sci. Sports Exerc..

[3] Larsen HB, Sheel AW (2015). The Kenyan runners. Scand. J. Med. Sci. Sports.

[4] Larsen HB, Christensen DL, Nolan T, Søndergaard H (2004). Body dimensions, exercise capacity and physical activity level of adolescent Nandi boys in western Kenya. Ann. Hum. Biol..

[5] Saltin B (1995). Aerobic exercise capacity at sea level and at altitude in Kenyan boys, junior and senior runners compared with Scandinavian runners. Scand. J. Med. Sci. Sports.

[6] Larsen, H. B. Kenyan dominance in distance running. *Comp. Biochem. Physiol. A: Mol. Integr. Physiol.* **136**, 161–170, doi:10.1016/s1095-6433(03)00227-7 (2003). 10.1016/s1095-6433(03)00227-7 14527638

[7] Lucia A (2006). Physiological characteristics of the best Eritrean runners-exceptional running economy. Appl. Physiol. Nutr. Metab..

[8] Lieberman DE (2010). Foot strike patterns and collision forces in habitually barefoot versus shod runners. Nature.

[9] Marino FE, Lambert MI, Noakes TD (2004). Superior performance of African runners in warm humid but not in cool environmental conditions. J. Appl. Physiol..

[10] Santos-Concejero J (2015). Gait-cycle characteristics and running economy in elite Eritrean and European runners. Int. J. Sports Physiol. Perform..

[11] Santos-Concejero J (2013). Differences in ground contact time explain the less efficient running economy in north African runners. Biol. Sport.

[12] Tam E (2012). Energetics of running in top-level marathon runners from Kenya. Eur. J. Appl. Physiol..

[13] Wishnizer RR, Inbar O, Klinman E, Fink G (2013). Physiological differences between Ethiopian and Caucasian distance runners and their effects on 10 km running performance. Adv Phys Educ.

[14] Shu Y (2015). Foot morphological difference between habitually shod and unshod runners. PLoS ONE.

[15] Sano K (2015). Can measures of muscle–tendon interaction improve our understanding of the superiority of Kenyan endurance runners?. Eur. J. Appl. Physiol..

[16] Patoz A, Lussiana T, Gindre C, Hébert-Losier K (2019). Recognition of foot strike pattern in Asian recreational runners. Sports.

[17] Andersen, J. J. & International Association of Athletics Federations. *The State of Running 2019,* (2020).

[18] Hawes MR (1994). Ethnic differences in forefoot shape and the determination of shoe comfort. Ergonomics.

[19] Zárate-Kalfópulos B, Romero-Vargas S, Otero-Cámara E, Correa VC, Reyes-Sánchez A (2012). Differences in pelvic parameters among Mexican, Caucasian, and Asian populations. J. Neurosurg. Spine.

[20] Sun P (2016). Autonomic recovery is delayed in Chinese compared with Caucasian following treadmill exercise. PLoS ONE.

[21] Chen W-L, O’Connor JJ, Radin EL (2003). A comparison of the gaits of Chinese and Caucasian women with particular reference to their heelstrike transients. Clin. Biomech..

[22] Gruber AH, Umberger BR, Braun B, Hamill J (2013). Economy and rate of carbohydrate oxidation during running with rearfoot and forefoot strike patterns. J. Appl. Physiol..

[23] Ogueta-Alday, A. N. A., RodrÍGuez-Marroyo, J. A. & GarcÍA-LÓPez, J. Rearfoot striking runners are more economical than midfoot strikers. *Med. Sci. Sports Exerc.* **46** (2014). 10.1249/MSS.0000000000000139 24002340

[24] Di Michele R, Merni F (2014). The concurrent effects of strike pattern and ground-contact time on running economy. J. Sci. Med. Sport.

[25] Støren, Ø., Helgerud, J. & Hoff, J. Running stride peak forces inversely determine running economy in elite runners. *J. Strength Cond. Res.* **25** (2011). 10.1519/JSC.0b013e3181b62c8a 20093965

[26] Paavolainen LM, Nummela AT, Rusko HK (1999). Neuromuscular characteristics and muscle power as determinants of 5-km running performance. Med. Sci. Sports Exerc..

[27] Williams KR, Cavanagh PR (1987). Relationship between distance running mechanics, running economy, and performance. J. Appl. Physiol..

[28] Lussiana T, Gindre C, Mourot L, Hébert-Losier K (2017). Do subjective assessments of running patterns reflect objective parameters?. Eur. J. Sport Sci..

[29] Harriss DJ, Macsween A, Atkinson G (2017). Standards for ethics in sport and exercise science research: 2018 update. Int. J. Sports Med..

[30] Oliveira AS, Pirscoveanu CI (2021). Implications of sample size and acquired number of steps to investigate running biomechanics. Sci. Rep..

[31] Svedenhag J, Sjödin B (1994). Body-mass-modified running economy and step length in elite male middle- and long-distance runners. Int. J. Sports Med..

[32] Tranberg R, Saari T, Zügner R, Kärrholm J (2011). Simultaneous measurements of knee motion using an optical tracking system and radiostereometric analysis (RSA). Acta Orthop..

[33] Hanavan E (1964). A mathematical model of the human body. AMRL-TR. Aerospace Med Res Lab.

[34] Dempster, W. T. *Space requirements of the seated operator: geometrical, kinematic, and mechanical aspects of the body with special reference to the limbs*. (Wright Air Development Center, 1955).

[35] Woltring H (1991). Representation and calculation of 3-D joint movement. Hum. Mov. Sci..

[36] Cole GK, Nigg BM, Ronsky JL, Yeadon MR (1993). Application of the joint coordinate system to three-dimensional joint attitude and movement representation: a standardization proposal. J. Biomech. Eng..

[37] Davis RB, Õunpuu S, Tyburski D, Gage JR (1991). A gait analysis data collection and reduction technique. Hum. Mov. Sci..

[38] Grood ES, Suntay WJ (1983). A joint coordinate system for the clinical description of three-dimensional motions: application to the knee. J. Biomech. Eng..

[39] Blankevoort L, Huiskes R, de Lange A (1988). The envelope of passive knee joint motion. J. Biomech..

[40] Kadaba MP, Ramakrishnan HK, Wootten ME (1990). Measurement of lower extremity kinematics during level walking. J. Orthop. Res..

[41] Piazza SJ, Cavanagh PR (2000). Measurement of the screw-home motion of the knee is sensitive to errors in axis alignment. J. Biomech..

[42] Altman AR, Davis IS (2012). A kinematic method for footstrike pattern detection in barefoot and shod runners. Gait Posture.

[43] Lussiana, T., Patoz, A., Gindre, C., Mourot, L. & Hébert-Losier, K. The implications of time on the ground on running economy: less is not always better. *J. Exp. Biol.* **222**, jeb192047, doi:10.1242/jeb.192047 (2019). 10.1242/jeb.192047 30787136

[44] Maiwald C, Sterzing T, Mayer TA, Milani TL (2009). Detecting foot-to-ground contact from kinematic data in running. Footwear Sci..

[45] Morin J-B, Dalleau G, Kyröläinen H, Jeannin T, Belli A (2005). A simple method for measuring stiffness during running. J. Appl. Biomech..

[46] Cohen, J. *Statistical Power Analysis for the Behavioral Sciences*. (Routledge, 1988).

[47] Hinkle, D. E., Wiersma, W. & Jurs, S. G. *Applied Statistics for the Behavioral Sciences*. 768 (Houghton Mifflin (p. 109), 2002).

[48] Sinclair J, Fau-Goodwin J, Richards J, Shore H (2016). The influence of minimalist and maximalist footwear on the kinetics and kinematics of running. Footwear Sci..

[49] Fuller JT, Bellenger CR, Thewlis D, Tsiros MD, Buckley JD (2015). The effect of footwear on running performance and running economy in distance runners. Sports Med..

[50] Sun X, Lam W-K, Zhang X, Wang J, Fu W (2020). Systematic review of the role of footwear constructions in running biomechanics: implications for running-related injury and performance. J. Sports Sci. Med..

[51] Hébert-Losier K (2020). Metabolic and performance responses of male runners wearing 3 types of footwear: Nike Vaporfly 4%, Saucony Endorphin racing flats, and their own shoes. J. Sport Health Sci..

[52] Tam, N., Tucker, R. & Astephen Wilson, J. L. Individual Responses to a barefoot running program: insight into risk of injury. *Am. J. Sports Med.* **44**, 777–784, doi:10.1177/0363546515620584 (2016). 10.1177/0363546515620584 26744483

[53] Tung KD, Franz JR, Kram R (2014). A test of the metabolic cost of cushioning hypothesis during unshod and shod running. Med. Sci. Sports Exerc..

[54] Van Alsenoy, K., van der Linden, M. L., Girard, O. & Santos, D. Increased footwear comfort is associated with improved running economy - a systematic review and meta-analysis. *Eur. J. Sport Sci.*, 1–13, doi:10.1080/17461391.2021.1998642 (2021). 10.1080/17461391.2021.1998642 34726119

[55] Roberts, T. J., Kram, R., Weyand, P. G. & Taylor, C. R. Energetics of bipedal running. I. Metabolic cost of generating force. *J. Exp. Biol.* **201**, 2745–2751, doi:10.1242/jeb.201.19.2745 (1998). 10.1242/jeb.201.19.2745 9732329

[56] Roberts, T. J., Chen, M. S. & Taylor, C. R. Energetics of bipedal running. II. Limb design and running mechanics. *J. Exp. Biol.* **201**, 2753–2762, doi:10.1242/jeb.201.19.2753 (1998). 10.1242/jeb.201.19.2753 9732330

[57] Kram R, Taylor CR (1990). Energetics of running: a new perspective. Nature.

[58] de Ruiter CJ, Verdijk PWL, Werker W, Zuidema MJ, de Haan A (2014). Stride frequency in relation to oxygen consumption in experienced and novice runners. Eur. J. Sport Sci..

[59] Quinn TJ, Dempsey SL, LaRoche DP, Mackenzie AM, Cook SB (2019). Step frequency training improves running economy in well-trained female runners. J. Strength Cond. Res..

[60] Kaneko, M., Matsumoto, M., Ito, A. & Fuchimoto, T. *Optimum step frequency in constant speed running*. (Human Kinetics, 1987).

[61] Tenforde AS, Borgstrom HE, Outerleys J, Davis IS (2019). Is cadence related to leg length and load rate?. J. Orthop. Sports Phys. Ther..

[62] Adams D, Pozzi F, Willy RW, Carrol A, Zeni J (2018). Altering cadence or vertical oscillation during running: effects on running related injury factors. Int. J. Sports Phys. Ther..

[63] Allen DJ, Heisler H, Mooney J, Kring R (2016). The effect of step rate manipulation on foot strike pattern of long distance runners. Int. J. Sports Phys. Ther..

[64] Nummela A, Keränen T, Mikkelsson L (2007). Factors related to top running speed and economy. Int. J. Sports Med..

[65] Lieberman DE, Warrener AG, Wang J, Castillo ER (2015). Effects of stride frequency and foot position at landing on braking force, hip torque, impact peak force and the metabolic cost of running in humans. J. Exp. Biol..

[66] Moore IS, Jones AM, Dixon SJ (2016). Reduced oxygen cost of running is related to alignment of the resultant GRF and leg axis vector: a pilot study. Scand. J. Med. Sci. Sports.

[67] Farley CT, González O (1996). Leg stiffness and stride frequency in human running. J. Biomech..

[68] Morin JB, Samozino P, Zameziati K, Belli A (2007). Effects of altered stride frequency and contact time on leg-spring behavior in human running. J. Biomech..

[69] Monte A, Muollo V, Nardello F, Zamparo P (2017). Sprint running: how changes in step frequency affect running mechanics and leg spring behaviour at maximal speed. J. Sports Sci..

[70] Saunders PU, Pyne DB, Telford RD, Hawley JA (2004). Factors affecting running economy in trained distance runners. Sports Med..

[71] Dickinson MH (2000). How animals move: an integrative view. Science.

[72] Jones AM (2006). The physiology of the world record holder for the women's marathon. Int. J. Sports Sci. Coach..

[73] Yue B (2011). Differences of knee anthropometry between Chinese and white men and women. J. Arthroplasty.

[74] Scholz MN, Bobbert MF, van Soest AJ, Clark JR, van Heerden J (2008). Running biomechanics: shorter heels, better economy. J. Exp. Biol..

[75] Hunter GR (2011). Tendon length and joint flexibility are related to running economy. Med. Sci. Sports Exerc..

[76] Ueno H (2018). Relationship between Achilles tendon length and running performance in well-trained male endurance runners. Scand. J. Med. Sci. Sports.

[77] Kunimasa Y (2014). Specific muscle-tendon architecture in elite Kenyan distance runners. Scand. J. Med. Sci. Sports.

[78] Mooses M (2015). Dissociation between running economy and running performance in elite Kenyan distance runners. J. Sports Sci..

[79] Hunter I (2019). Running economy, mechanics, and marathon racing shoes. J. Sports Sci..

[80] Sloan RS, Wight JT, Hooper DR, Garman JEJ, Pujalte GGA (2020). Metabolic testing does not alter distance running lower body sagittal kinematics. Gait Posture.

