# Supplementary material for: Non-South East Asians have a better running economy and different anthropometrics and biomechanics than South East Asians
Source: Sci Rep. 2022 Apr 15;12:6291. doi: 10.1038/s41598-022-10030-4 (PMC9012817; doi:10.1038/s41598-022-10030-4)
Supplement: Supplementary file 1 — Supplementary Information. [file 41598_2022_10030_MOESM1_ESM.docx]

*Supplementary Materials for:*

**Non-South East Asians have a better running economy and different anthropometrics and biomechanics than South East Asians.**

**S1. Running economy between South East Asians and non-South East Asians expressed in ml/kg/km**

Expressing running economy (RE) in ml/kg/km resulted in larger differences between South East Asians (SEA) and non-SEA runners than in the main manuscript. SEA runners were significantly less economical (10%) than non-SEA runners (average across speeds: 191.7 ± 14.9 vs 171.9 ± 10.8 ml/kg/km), with a *large* main effect of group on RE (*p* < 0.001, *large* effect size: *d* = 1.52; Figure S1). There were no significant main effect of speed (*p* = 0.28) and group x speed interaction effect (*p* = 0.89) on RE.

**Figure S1.** Running Economy (RE; in ml/kg/km) of South East Asian (SEA) and non-South East Asian (non-SEA) runners at several endurance running speeds. Linear mixed effects modelling identified a significant group effect (p ≤ 0.05).

Relationships between RE and anthropometric characteristics unrelated to other anthropometric characteristics (mass and ratio of leg length over height) were computed and reported in Table S1. All correlations were negligible or low, and non-significant.

**Table S1.** Pearson correlation coefficients between running economy (in ml/kg/km) and anthropometric characteristics (mass and ratio of leg length over height), together with their corresponding p-values underneath for South East Asian (SEA), non-South East Asian (non-SEA), as well as all runners pooled together (ALL). Statistical significances (p ≤ 0.05) are indicated in bold.

| Group | SEA | | | non-SEA | | | ALL | | |
| --- | --- | --- | --- | --- | --- | --- | --- | --- | --- |
| Running speed (km/h) | 10 | 12 | 14 | 10 | 12 | 14 | 10 | 12 | 14 |
| Mass (kg) | 0.26  0.32 | 0.27  0.29 | -0.06  0.82 | 0.47  0.06 | 0.35  0.17 | 0.16  0.54 | -0.06  0.74 | -0.13  0.46 | -0.29  0.10 |
| Leg length over  height (%) | 0.01  0.98 | -0.17  0.53 | -0.07  0.80 | 0.20  0.44 | 0.05  0.86 | -0.04  0.87 | 0.00  0.99 | -0.13  0.46 | -0.12  0.50 |

*Note.* Only the relationships between running economy and mass and ratio of leg length over height were considered because mass was highly and significantly correlated to height, leg length, and body mass index.

For SEA runners, a *high* positive correlation was seen between RE and contact time (*t_c_*) at 12 km/h (|*r*| ≥ 0.76, *p* ≤ 0.001; Table S1). Step frequency (SF) and ankle angle ($\theta_{\mathrm{ankle}}$) at footstrike at 10 km/h were *moderately* and negatively correlated to RE, whereas step length (SL) was *moderately* and positively correlated to RE at 10 km/h (|*r*| ≥ 0.54, *p* ≤ 0.02; Table S1). At 12 km/h, SF, *k*_leg_, and $\theta_{\mathrm{ankle}}$ at footstrike and toe-off were *moderately* and negatively correlated to RE, whereas SL was *moderately* and positively correlated to RE (|*r*| ≥ 0.54, *p* ≤ 0.03; Table S1).

For non-SEA runners, there was no *moderate* or *high* correlations between RE and any biomechanical variables.

For runners combined, footstrike angle (FSA) and $\theta_{\mathrm{ankle}}$ at footstrike were *moderately* and negatively correlated to RE at 10 and 12 km/h ((|*r*| ≥ 0.50, *p* ≤ 0.003; Table S1). Table S1 presents all correlations, including the *low*, *negligible*, and non-significant ones.

**Table S2.** Pearson correlation coefficients between running economy (in ml/kg/km) and biomechanical variables [step frequency (SF), step length (SL), contact time (t_c_), flight time (t_f_), spring-mass characteristics of the lower limb as given by leg stiffness (k_leg_), footstrike angle (FSA), and flexion-extension ankle ($\theta_{ankle}$) and knee ($\theta_{knee}$) joint angle at footstrike (FS) and toe-off (TO)], together with their corresponding p-values underneath for South East Asian (SEA), non-South East Asian (non-SEA), as well as all runners pooled together (ALL). Statistical significances (p ≤ 0.05) are indicated in bold. Gray shaded boxes denote correlation coefficients above an absolute value of 0.5 (moderate). SL was expressed as a percentage of participant's leg length in addition to raw units.

| Group | SEA | | | non-SEA | | | ALL | | |
| --- | --- | --- | --- | --- | --- | --- | --- | --- | --- |
| Running speed (km/h) | 10 | 12 | 14 | 10 | 12 | 14 | 10 | 12 | 14 |
| SF (steps/min) | -0.68 **0.003** | -0.66  **0.004** | -0.11  0.67 | -0.20  0.43 | -0.22  0.40 | -0.12  0.65 | -0.10  057 | -0.07  0.70 | 0.14  0.42 |
| SL (cm) | 0.69  **0.002** | 0.67  **0.003** | 0.13  0.63 | 0.23  0.38 | 0.21  0.41 | 0.10  0.69 | 0.10  0.56 | 0.07  0.70 | -0.13  0.46 |
| SL (%)^a^ | 0.29  0.26 | 0.38  0.14 | 0.09  0.72 | -0.35  0.17 | -0.17  0.50 | -0.11  0.67 | 0.05  0.76 | 0.15  0.39 | 0.06  0.74 |
| *t_c_* (ms) | 0.25  0.34 | 0.78  **<0.001** | 0.29  0.27 | -0.06  0.82 | 0.28  0.28 | 0.41  0.10 | -0.11  0.52 | 0.18  0.32 | 0.01  0.94 |
| *t_f_* (ms) | 0.10  0.72 | -0.25  0.33 | -0.21  0.43 | 0.26  0.32 | -0.02  0.95 | -0.18  0.48 | 0.05  0.77 | -0.14  0.43 | -0.16  0.36 |
| *k*_leg_ (kN/m) | -0.26  0.31 | -0.54  **0.03** | -0.38  0.13 | 0.38  0.14 | 0.11  0.69 | -0.07  0.78 | -0.12  0.51 | -0.30  0.09 | -0.28  0.11 |
| FSA (°) | -0.42  0.10 | -0.38  0.13 | -0.06  0.81 | 0.05  0.85 | 0.02  0.94 | -0.11  0.66 | -0.50  **0.003** | -0.52  **0.001** | -0.45  **0.007** |
| $\theta_{\mathrm{ankle}}$ at FS (°) | -0.54  **0.02** | -0.55  **0.02** | -0.07  0.78 | -0.12  0.66 | 0.00  1.00 | -0.09  0.74 | -0.54  **<0.001** | -0.51  **0.002** | -0.38  **0.03** |
| $\theta_{\mathrm{ankle}}$ at TO (°) | -0.30  0.25 | -0.62  **0.008** | -0.24  0.35 | 0.00  0.99 | -0.07  0.78 | -0.31  0.23 | -0.33  0.06 | -0.48  **0.004** | -0.32  0.06 |
| $\theta_{\mathrm{knee}}$ at FS (°) | 0.01  0.98 | 0.04  0.89 | 0.00  1.00 | -0.08  0.76 | 0.08  0.77 | 0.13  0.63 | -0.12  0.48 | -0.03  0.84 | 0.03  0.87 |
| $\theta_{\mathrm{knee}}$ at TO (°) | -0.01  0.97 | 0.03  0.90 | 0.18  0.50 | 0.16  0.53 | 0.30  0.25 | 0.31  0.23 | 0.23  0.20 | 0.35  **0.04** | 0.43  **0.01** |

^a^ Step length normalized to leg length.

**S2. Morphological factors potentially explaining differences in running economy between** **South East Asians and non-South East Asians**

Asian and non-Asian individuals have been shown to differ morphologically^1-3^. For instance, the shape of the forefoot of Japanese and Korean males differs from North American males^1^; Chinese knees (mediolateral and anteroposterior size of the femur) are generally smaller than Caucasian ones^2^; and pelvic parameters (e.g., pelvic tilt and incidence) also differ between Asian, Mexican, and Caucasian individuals^3^. These specific parameters were not examined and, although most likely not associated with RE directly, can potentially play a role in the biomechanical and physiological differences we observed.

Achilles tendon moment arms and foot-lever ratios are two additional parameters that relate to RE. Indeed, a previous study found a strong correlation between the moment arm of the Achilles tendon and RE, where smaller muscle moment arms correlated with lower rates of metabolic energy consumption^4^. Hunter et al.^5^ also observed that longer lower limb tendons (especially Achilles tendon) and less flexible lower limb joints were linked with improved RE. Recently, Ueno et al.^6^ proposed that longer Achilles tendons may be advantageous to achieve superior running endurance performance associated with better RE, in support of previous findings. In addition, longer moment arms and shorter feet (smaller foot-lever ratio) of elite Kenyan than Japanese runners were associated with better RE^7^. However, discrepancies exist in the scientific literature, as smaller moment arms have been associated with greater RE in high-level Kenyan distance runners^8^. Clearly, more research is needed on this subject as these parameters were not measured in this study, but could have partly explained differences in RE between SEA and non-SEA runners.

**References**

1 Hawes, M. R. *et al.* Ethnic differences in forefoot shape and the determination of shoe comfort. *Ergonomics* **37**, 187-196, doi:10.1080/00140139408963637 (1994).

2 Yue, B. *et al.* Differences of knee anthropometry between Chinese and white men and women. *J. Arthroplasty* **26**, 124-130, doi:10.1016/j.arth.2009.11.020 (2011).

3 Zárate-Kalfópulos, B., Romero-Vargas, S., Otero-Cámara, E., Correa, V. C. & Reyes-Sánchez, A. Differences in pelvic parameters among Mexican, Caucasian, and Asian populations. *J. Neurosurg. Spine* **16**, 516-519, doi:10.3171/2012.2.Spine11755 (2012).

4 Scholz, M. N., Bobbert, M. F., van Soest, A. J., Clark, J. R. & van Heerden, J. Running biomechanics: shorter heels, better economy. *J. Exp. Biol.* **211**, 3266, doi:10.1242/jeb.018812 (2008).

5 Hunter, G. R. *et al.* Tendon length and joint flexibility are related to running economy. *Med. Sci. Sports Exerc.* **43**, 1492-1499, doi:10.1249/MSS.0b013e318210464a (2011).

6 Ueno, H. *et al.* Relationship between Achilles tendon length and running performance in well-trained male endurance runners. *Scand. J. Med. Sci. Sports* **28**, 446-451, doi:10.1111/sms.12940 (2018).

7 Kunimasa, Y. *et al.* Specific muscle-tendon architecture in elite Kenyan distance runners. *Scand. J. Med. Sci. Sports* **24**, e269-274, doi:10.1111/sms.12161 (2014).

8 Mooses, M. *et al.* Dissociation between running economy and running performance in elite Kenyan distance runners. *J. Sports Sci.* **33**, 136-144, doi:10.1080/02640414.2014.926384 (2015).
